# Multimodal Elimination for Intoxication with a Lethal Dose of Organic Mercury

**DOI:** 10.1155/2019/4275918

**Published:** 2019-01-16

**Authors:** L. C. Napp, C. Moelgen, F. Wegner, P. Heitland, H. D. Koester, M. Klintschar, M. Hiss, A. Schaper, B. Schieffer, J. Bauersachs, A. Schäfer, J. Tongers

**Affiliations:** ^1^Department of Cardiology and Angiology, Hannover Medical School, Germany; ^2^Department of Pneumology, Hannover Medical School, Germany; ^3^Department of Neurology, Hannover Medical School, Germany; ^4^Medical Laboratory Bremen, Bremen, Germany; ^5^Department of Legal Medicine, Hannover Medical School, Germany; ^6^Department of Nephrology, Hannover Medical School, Hannover, Germany; ^7^GIZ-Nord Poisons Centre, University Medical Centre Göttingen, Germany; ^8^Department of Cardiology, University Hospital Marburg, Germany

## Abstract

We here report on a case of massive organic mercury intoxication in a 40-year-old man that resulted in progressive multiorgan failure. We treated the patient intravenously and enterally with the chelating agent (RS)-2,3-bis(sulfanyl) propane-1-sulfonic acid (DMPS) in addition to hemodialysis. The patient was treated for 6 weeks and could successfully be weaned from mechanical ventilation and hemodialysis. He awoke and was sent to rehabilitation, but unfortunately died 7 months later from refractory status epilepticus. Autopsy revealed severe brain atrophy consistent with organ damage from massive mercury intoxication. The present case illustrates that bimodal DMPS application is sufficient for detoxification from lethal mercury levels, with an associated chance for weaning of organ support and survival to discharge. The case further reminds us of intoxication as a cause of multiorgan dysfunction. We propose to immediately initiate combined parenteral and enteral detoxification in cases of methyl mercury intoxication, especially in cases of high doses.

## 1. Introduction

Intoxications are often dangerous if not fatal conditions, but their identification is usually difficult. Delayed diagnosis may in turn result in irreversible organ failure or death. In the present case, rigorous differential diagnosis revealed massive mercury intoxication as the cause of the patient's clinical signs and symptoms and allowed for specific therapy.

## 2. Case Presentation

A previously healthy 40-year-old man was referred to our intensive care unit from a regional hospital with aphasia, somnolence, weakness, maculopapular exanthema with palmoplantar hyperkeratosis and renal failure. He had been suffering from progressive fatigue and weakness for several weeks. On admission to the ICU the patient was somnolent, only responding with undirected movements to painful stimuli and incomprehensible sounds. Communication was not possible. Ptosis was evident, but pupils were reactive with normal accommodation to light. Severe tetraparesis (legs > arms) was present, and the patient was hardly able to move his tongue. Muscle fasciculations were apparent, reflexes on arms and legs were nearly absent, and Babinski's sign was positive. The patient had an initial heart rate of 103 bpm (sinus rhythm) and a blood pressure of 150/90 mmHg in the presence of fever.

Sepsis was unlikely due to high diastolic blood pressure and nearly normal parameters of inflammation. A parainfectious syndrome was also unlikely due to normal antibody profiling and complement activities. Ultrasound revealed hepatosplenomegaly and enlarged and swollen kidneys with compacted marrow and echogenic cortex. Renal biopsy showed nonpurulent interstitial nephritis. Skin biopsy demonstrated perivascular dermatitis. Magnetic resonance imaging and lumbar puncture showed no signs of myelitis, encephalitis, and meningitis. Electroneurography and -myography demonstrated reduced nerve conduction velocity and spontaneous activity, consistent with severe axonal polyneuropathy. Thus, we suspected axonal Guillain-Barré syndrome and performed plasma exchange and immunoglobulin therapy. However the patient's condition further deteriorated. Tetraplegia occurred, and the patient developed progressive weakness of the respiratory muscles and coma, for which intubation and mechanical ventilation had to be started.

**Figure 1:**
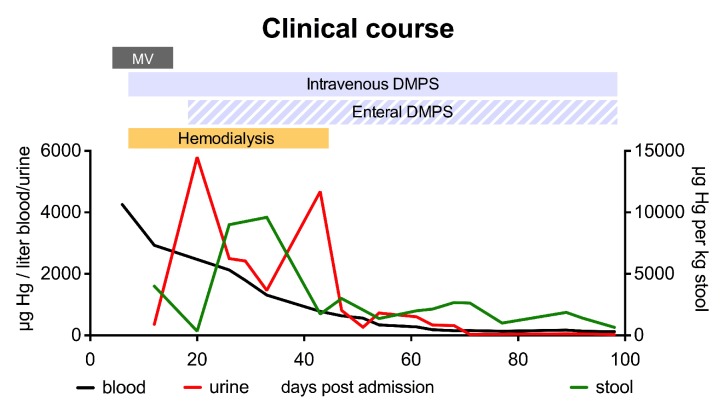
**Clinical course after mercury intoxication. **The chelating agent (RS)-2,3-bis(sulfanyl) propane-1-sulfonic acid (DMPS) was given enterally and parenterally for elimination. Black line: peripheral blood (left y axis, EDTA whole blood), red line: urine (left y axis), green line: stool (right y axis). Hg: hydrargium/mercury, MV: mechanical ventilation.

**Figure 2:**
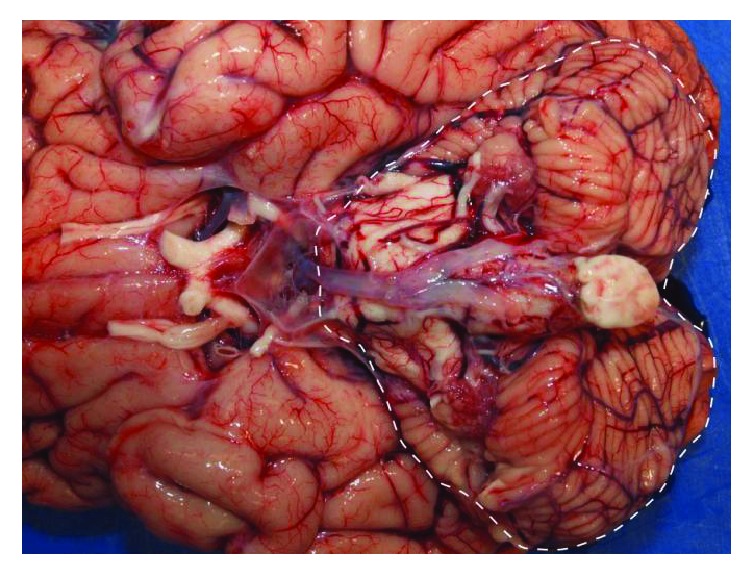
**Autopsy result.** Picture of the medulla oblongata, the cerebellum, and parts of the cerebrum as seen from caudal. The veins are very prominent, and the superficial structures especially of the cerebellum and the pons (dashed line) show marked atrophy, which is a classic late sign of mercury intoxication.

Due to deterioration on therapy we questioned our diagnosis. The broad clinical picture involving skin, kidneys, and the nervous system could also be caused by intoxication. While levels of many other compounds tested were normal, mercury levels were exceedingly high in peripheral blood (4255 *μ*g/l, Figure 1, Table 1). Chemical analysis confirmed predominant presence of methyl mercury in blood, suggesting intoxication with organic mercury (Supplementary Materials (available here)). Despite extensive history taking and investigation, also of the social and occupational environment, the definite source of intoxication remained elusive. In retrospect, clinical signs and symptoms were consistent with severe organic mercury intoxication. Intravenous administration of the chelating agent (RS)-2,3-bis(sulfanyl) propane-1-sulfonic acid (DMPS) was combined with hemodialysis to eliminate complexed mercury. This resulted in a strong reduction of mercury levels over time (Figure 1). As methyl mercury is present in the gut of intoxicated patients and absorbed via an enterohepatic circuit [1], we added enteral DMPS to further enhance elimination. This bimodal chelating therapy was associated with a strong decline of blood mercury levels. In parallel mercury levels in urine and stool increased, demonstrating efficient detoxification and supporting the concept of bimodal mercury elimination. Despite the challenging diagnosis and delay in detoxification as well as the exceedingly high mercury levels, elimination was associated with improvement of clinical symptoms and organ functions. The patient gradually regained vigilance as well as motoric and neural functions. He was also successfully weaned from mechanical ventilation and hemodialysis (Figure 1). A detailed description of clinical recovery is provided in Table 2. Arrhythmias did not occur during hospitalization. Eight weeks after admission to our hospital, the awake patient was sent to a rehabilitation facility. After 3 months of continuous DMPS treatment concentrations of mercury in EDTA whole blood, serum, and urine were 122, 24, and 24 *μ*g/L, respectively. Unfortunately, the patient died 7 months after discharge from our hospital from refractory status epilepticus. Autopsy revealed severe atrophy of cerebellum, pons, and medulla oblongata (Figure 2), findings that are common after severe mercury intoxication [2].

## 3. Discussion

Mercury exposure usually occurs through ingestion of contaminated fish, contact to seed mordant, fungicides, merbromin solution (an antiseptic agent), vapour from broken thermometers [3], or occupational contact. Of all occurring forms, organic mercury has the highest toxicity with a total body half-life of 50 days. Prominent examples of organic mercury intoxication are Minamata disease [4] and the case of the American chemist Karen Wetterhahn [2]. In the general population the mean blood mercury level is 1.7 *μ*g/l [5]. While levels below 10 *μ*g/l in blood are usually nontoxic, sensitive individuals start to develop symptoms from 35 *μ*g/l on. Levels above 200-300 *μ*g/l are thought to be fatal if left untreated. In our patient initial total mercury concentration in EDTA whole blood was 4255 *μ*g/l. Shortly after start of DMPS, total mercury levels in whole blood and serum were 2929 and 899 *μ*g/L, respectively. At the same time, the concentration of methyl mercury in EDTA whole blood was 1538 *μ*g/L as determined by headspace capillary gas chromatography mass spectrometry. Thus, the majority of mercury was found intracellularly and to be methyl mercury, with a very high mercury concentration in hair samples (Table 1), all of which supported and confirmed intoxication with organic mercury.

Although treatment with a chelating agent effectively lowers blood levels of mercury, its benefit on brain levels and damage is rather limited, especially in the case of methyl mercury [6]. With delayed onset of therapy, the chance to prevent sustained pathological effects is lower compared to immediate onset [7]. As such, our case demonstrates that delayed therapy of intoxication with a lethal dose of mercury is feasible and efficiently lowers blood levels. Regarding the excessive levels of mercury in our patient, we were impressed that the patient survived at all and could be sent to rehabilitation awake without organ support.

Overall, we believe that combined enteral and parenteral treatment with DMPS is beneficial in order to enhance elimination of methyl mercury by interruption of the enterohepatic circulation.

## Figures and Tables

**Table 1:** **Levels of drugs or compounds tested in different compartments.** Abnormal values are highlighted with bold letters.

**Toxicology**			
**Compound or drug**	**Specimen**	**Patient level**	**Normal value**

**Initial measurements**			
Amphetamine & derivates	urine	not detectable	
Barbiturates	urine	not detectable	
Benzodiazepines	urine	not detectable	
Buprenorphine	urine	not detectable	
Cocaine metabolites	urine	not detectable	
Morphine & Derivates	urine	not detectable	
Phenothiazine	urine	not detectable	
Tetrahydrocannabinol derivates	urine	not detectable	
Cadmium	EDTA whole blood	< 0.2 *μ*g/l	< 1.0 *μ*g/l
Copper	serum	15.6 *μ*mol/l	11.0 - 22.0 *μ*mol/l
Lead	EDTA whole blood	12 *μ*g/l	< 120 *μ*g/l
Zinc	serum	92 *μ*g/dl	70 - 150 *μ*g/dl
Mercury	EDTA whole blood	**4255 ** ***μ*** **g/l**	< 2.0 *μ*g/l
Arsine	urine	1.4 *μ*g/l	< 25.0 *μ*g/l
Pentachlorophenol	urine	< 0.30 *μ*g/l	< 5.0 *μ*g/l
Thallium	EDTA whole blood	< 0.2 *μ*g/l	< 0.6 *μ*g/l
Thallium	urine	< 0.2 *μ*g/l	< 0.7 *μ*g/l

**Compartment measurements**^**∗**^			
Mercury	EDTA whole blood	**2929 ** ***μ*** **g/l**	< 2.0 *μ*g/l
Methyl mercury	EDTA whole blood	**1538.0 ** ***μ*** **g/l**	< 1.0 *μ*g/l
Mercury	serum	**899 ** ***μ*** **g/l**	< 2.0 *μ*g/l
Mercury	urine	**360 ** ***μ*** **g/l**	< 1.0 *μ*g/l
Mercury	hair	**713 ** ***μ*** **g/g**	< 2.0 *μ*g/g

^*∗*^: after start of treatment with DMPS.

**Table 2:** **Clinical course during detoxification with DMPS.**

**Clinical Course**	
**Day** ^**∗**^	**Observation**

3	pupils beginning to react to light
4	short periods of assisted ventilation
7	muscle fasciculations absent
9	spontaneous eye opening
10	spontaneous breathing
11	exanthema starts to decline
14	continuous spontaneous breathing
15	eye opening to verbal command
16	change from CRRT to intermittent dialysis
17	increasing vigilance
19	patient startles upon verbal command reflexes start to return
20	first spontaneous movement of extremities
23	first successful mobilization (with assistance) exanthema diminishes
25	increasing extremity movement
30	switch from dialysis to ultrafiltration only
31	visual fixation
33	patient mobilized from bed to chair
34	all standard reflexes bilaterally reestablished
37	spontaneous directed movement of all extremities

## Data Availability

The data used to support the findings of this study are included within the article. Further data are restricted by institutional policies in order to protect patient privacy and therefore cannot be made publicly available.

## Supplementary Materials

Supplementary Materials Supplementary Methods. Description of laboratory methods used for mercury measurement.
