# Supplementary material for: Multimodal Elimination for Intoxication with a Lethal Dose of Organic Mercury
Source: Case Rep Crit Care. 2019 Jan 16;2019:4275918. doi: 10.1155/2019/4275918 (PMC6354150; doi:10.1155/2019/4275918)
Supplement: Supplementary Materials — Supplementary Methods. Description of laboratory methods used for mercury measurement. [file 4275918.f1.docx]

*Multimodal Elimination For Intoxication*

*With A Lethal Dose Of Organic Mercury*

L. C. Napp^1, §^, C. Moelgen ^2^, F. Wegner^3^, P. Heitland^4^, H. D. Koester^4^, Klintschar M^5^, A. Schaper^6^, B. Schieffer^7^, J. Bauersachs^1^, A. Schäfer^1^, J. Tongers^1^

1: Department of Cardiology and Angiology, Hannover Medical School, Germany

2: Department of Pneumology, Hannover Medical School, Germany

3: Department of Neurology, Hannover Medical School, Germany

4: Medical Laboratory Bremen, Bremen, Germany

5: Department of Legal Medicine, Hannover Medical School, Germany

6: GIZ-Nord Poisons Centre, University Medical Centre Göttingen, Germany

7: Department of Cardiology, University Hospital Marburg, Germany

§: correspondence to napp.christian@mh-hannover.de

**Supplementary Methods**

Total mercury was determined by cold-vapor atomic fluorescence spectrometry (CV-AFS) applying a Mercur AF-spectrometer with flow injection (Analytik Jena, Jena, Germany). For sample preparation 500 µL (blood, urine) or 500 mg (stool) were dissolved with 1 mL of a solution containing 10 g potassium peroxodisulfate, 25 ml concentrated sulphuric acid and 200 mL HNO_3_ concentrated nitric acid. Calibration was performed using matrix-matched calibration solutions. Precision and accuracy of the results were investigated using control materials from Recipe (Munich, Germany). The AFS method is regularly applied to participate in national and international intercomparison programmes for the Hg determination in blood and urine. As a reference method we also applied inductively coupled plasma mass spectrometry (ICP-MS) for total mercury determination to confirm accuracy of the results. In this case we used an Agilent 7700x ICP-MS spectrometer (Agilent Technologies, Waldbronn, Germany). Methylmercury in blood was determined by headspace capillary gas chromatography mass spectrometry (GC-MS).(1)

**Supplemental References**

1. Hippler J, Hoppe HW, Mosel F, et al. Comparative determination of methyl mercury in whole blood samples using GC-ICP-MS and GC-MS techniques. J Chromatogr B Analyt Technol Biomed Life Sci 2009;877(24):2465-2470.
